# Investigation of the Association between Metabolic Syndrome and Disease Activity in Rheumatoid Arthritis

**DOI:** 10.1100/tsw.2011.111

**Published:** 2011-06-09

**Authors:** Maryam Sahebari, Ladan Goshayeshi, Zahra Mirfeizi, Zahra Rezaieyazdi, Mohammad R. Hatef, Majid Ghayour-Mobarhan, Saeed Akhlaghi, Amirhossein Sahebkar, Gordon A. Ferns

**Affiliations:** ^1^ Rheumatic Diseases Research Center (RDRC), Mashhad University of Medical Sciences, Mashhad, Iran; ^2^ Cardiovascular Research Center, Avicenna Research Institute, Mashhad University of Medical Sciences, Mashhad, Iran; ^3^ Biochemistry and Nutrition Research Center, Avicenna Research Institute, Mashhad University of Medical Sciences, Mashhad, Iran; ^4^ Deputy of Research, Faculty of Medicine, Mashhad University of Medical Sciences, Mashhad, Iran; ^5^ Biotechnology Research Center and School of Pharmacy, Mashhad University of Medical Sciences, Mashhad, Iran; ^6^ Institute for Science and Technology in Medicine, University of Keele, Guy Hilton Research Centre, Stoke on Trent, Staffordshire, UK

**Keywords:** rheumatoid arthritis, metabolic syndrome, body mass index, disease activity score

## Abstract

Rheumatoid arthritis (RA) is the most common form of autoimmune arthritis. Increased prevalence of metabolic syndrome (MetS) in RA may occur secondary to specific drug treatment and reduced physical activity associated with this condition. However, some recent studies suggest contradictory theories about the association of RA with MetS. This study was designed to evaluate the frequency of MetS in RA patients and the relationship between MetS with RA disease activity and body mass index (BMI). The study was conducted on 120 RA patients and 431 age- and sex-matched apparently healthy controls. A considerable proportion of patients were being treated with prednisolone and/or methotrexate and/or hydroxychloroquine. Disease activity was measured by the 28 joint count of disease activity score-Cerythrocyte sedimentation rate (DAS28ESR). MetS was evaluated according to International Diabetic Federation (IDF) and Adult Treatment Panel III (ATP III) criteria. The prevalence of MetS was significantly higher in the control group (*p* = 0.005). We did not find any difference in the prevalence of MetS between the patients with DAS < 3.2 and DAS ≥ 3.2. There was no association between the DAS28 score and the presence of MetS components by either definition. Multiple logistic regression analysis showed that the odds of a DAS > 3.2 in patients with BMI between 25 and 30 kg/m_2_ (OR = 0.1, *p* = 0.01) and BMI > 30 kg/m_2_ (OR = 0.3, *p* = 0.1), in comparison to BMI < 25 kg/m_2_, was 1/5 and 1/3, respectively. RA was not found to increase the risk of MetS. In addition, disease activity in RA patients was not influenced by the presence of MetS.

### INTRODUCTION

Rheumatoid arthritis (RA) is an autoimmune disease associated with an increased risk of cardiovascular diseases (CVD) that accounts for about half of all deaths in this population[1]. Several cardiovascular risk factors are present at higher frequencies in patients with RA. In addition, chronic inflammation, drugs used in treatment strategies, and sedentary life style may play an important role in increasing the risk of CVD in RA[2,3]. The prevalence of metabolic syndrome (MetS) and coronary artery disease (CAD) are all increasing globally, especially in the Middle East[4]. Until recently, it was believed that MetS and its components are important risk factors for CVD in different populations[5,6], but some authors have suggested that the proposed definitions of MetS are not applicable to an Iranian population[7]. Sattar et al. have also proposed that MetS and its components have weak or no association with vascular risk in elderly populations[8]. A study among Mediterranean RA patients failed to show a significant difference between the prevalence of MetS in RA patients and the local general population serving as controls[9]. However, the reported prevalence of MetS varies across populations. This may be due to a number of population differences, including the age of the samples investigated, multiple genetic variations, environmental factors, and ethnicity of the populations studied. Furthermore, different diagnostic criteria have been applied[9,10]. The criteria for MetS have evolved since the first definition by the World Health Organization (WHO)[11]. More than ten definitions of MetS are now extant; among them, those from the WHO[11], Adult Treatment Panel III (ATP III)[12], and International Diabetic Federation (IDF)[13] are the most important for clinical use. The major features of MetS include hyperglycemia, hypertriglyceridemia, low level of high-density lipoprotein cholesterol (HDL-C), hypertension, and central obesity[9,13]. Although RA patients have several risk factors for CVD[14,15], it is unclear whether MetS is more prevalent in RA patients than the normal population or if it is an important predictor of CVD for these patients in all races[8]. It is clear that physical inactivity may lead to MetS and several of its components. RA patients have different risk factors for sedentary life style, especially patients with active arthritis or with deformities in the lower limb joints. Previous data have shown that corticosteroids used in the treatment of RA may be associated with the development of diabetes and hypertension[16]. Other glucocorticoids may have insulin-sensitizing effects in short-term therapy and recent data suggest that low-dose glucocorticoid therapy in RA patients may change the lipid profile favorably[17,18]. Furthermore, high serum levels of the inflammatory cytokines TNF$\alpha$ and IL-1 are often observed in RA patients, and may be associated with weight loss and cachexia[19]. Hydroxychloroquine sulfate, a disease-modifying antirheumatic drug (DMARD) that is prescribed for the treatment of RA, has lipid- and glucose-lowering effects[20,21]. It has also been reported that methotrexate therapy decreases the prevalence of MetS in RA patients[22]. RA patients may also visit physicians regularly and, consequently, may be more advised to exercise regularly, have weight loss programs, and control hyperlipidemia and diabetes, in addition to aggressive control of inflammation, which may therefore affect the prevalence of components of MetS[23]. In other words, the frequency of MetS in RA patients is influenced not only by traditional factors such as race, age, and dietary customs, but also by disease-specific factors[8,14]. To this end, we suggested an assessment of the frequency of MetS in a cohort of patients with RA, a comparison of the frequency of this syndrome with ATP III and IDF definitions, and an exploration of the possible relationship of MetS, its components, and body mass index (BMI) with disease activity in the Iranian population.

### METHODS

#### Subjects

One hundred and twenty RA patients, according to the 1987 American College of Rheumatology (ACR) criteria, including 106 (88.3%) females and 14 (11.7%) males, participated in this case-control study. The patients had different stages of disease activity, assessed by the disease activity score in 28 joints–erythrocyte sedimentation rate (DAS28ESR)[24]. Five hundred age-, sex-, and race-matched apparently healthy volunteers (431 [86.2%] women and 69 [13.8%] men) from urban and rural residences of the Great Khorasan province in Iran served as the control group in the present study. These control samples were selected from those of a previous MetS cohort[7].

At baseline, the volunteers' demographic, anthropometric, clinical, and laboratory data were collected for detection of MetS, according to both IDF and National Cholesterol Education Program (NCEP)–ATP III criteria.

All participants gave informed written consent to participate in the study, which was approved by the Ethics Committee of Mashhad University of Medical Sciences.

### Anthropometric Measurements

Anthropometric parameters, including height, weight, waist circumference, and BMI, as well as systolic and diastolic blood pressures, were measured for all participants as described previously[25].

### Biochemical Measurements

A fasting blood sugar (FBS), erythrocyte sedimentation rate (ESR), lipid profile (including total cholesterol, triglycerides [TG], HDL-C, and low-density lipoprotein cholesterol [LDL-C]), was determined for each participant after an overnight fasting. After being allowed to clot, the blood was then centrifuged at 2500 rpm for 15 min at room temperature to obtain serum. Hemolyzed samples were excluded from analysis. Serum was stored at −20°C prior to analysis. Serum FBS and lipid profile were measured by enzymatic methods. Their assessment also included a structured interview, laboratory tests, review of medical records and smoking habits (including cigarettes and hookah), and personal or family history of ischemic heart disease.

### Assessment of RA Disease Activity

Patients were selected according to the ACR criteria for RA[26], and their disease activity was measured with the DAS28ESR[24] by a regular program of physical examination and evaluation of visual analogues scale (VAS), with a 100-cm ruler, by a single rheumatologist, in the morning between 8 and 10 a.m.

### Statistical Analysis

SPSS software (version 11.5, Chicago, IL, USA) was used for statistical analysis. The Kolomogrov-Smirnov test was used to evaluate the normality of the data. Values were expressed as mean ± SD for normally distributed variables and median with interquartile range (IQR) for non-normally distributed data. Baseline demographics and clinical characteristics were compared between groups using independent samples *t*-test, one-way analysis of variance (ANOVA), Chi-square and/or Fisher's exact test, as appropriate. Bivariate correlations were assessed using Pearson's and Spearman's correlation coefficients for normally and non-normally distributed data, respectively. The relationship between the presence of MetS or its components and RA disease activity (according to the DAS28ESR) was assessed by multivariate logistic regression analysis. Linear logistic regression analysis was used to assess the relationship between demographics and MetS components.

## RESULTS

### Demographic Data

One hundred and twenty patients (106 women and 14 men) and 500 apparently healthy controls (431 women and 69 men) participated in this study. There was no significant difference between the groups regarding gender or age. Demographic characteristics of case and control groups are shown in Table 1. Table 2 outlines the frequency of metabolic components in the patient and control groups.

**TABLE 1**
**Demographic Data of Patient and Control Groups**

| Demographics and Metabolic Components | Patients (n = 120) | Female (n = 106) | Male (n = 14) | Controls (n = 500) | Female (n = 431) | Male (n = 69) | *p* Value (Patients vs. Controls) |
|---|---|---|---|---|---|---|---|
| | Mean ± SD | | | | | | |
| Age (years) | 45.5 ± 13 | 45.3 ± 13 | 46.5 ± 16 | 45.6 ± 12 | 44.7 ± 12 | 44.1 ± 13 | 0.5 |
| Systolic blood pressure (mmHg)* | 115 ± 19 | 116 ± 19 | 112 ± 13 | 128.2 ± 23 | 127.7 ± 23 | 130 ± 23 | 0.0001** |
| Diastolic blood pressure (mmHg)* | 75.1 ± 9 | 75.5 ± 9 | 72 ± 9 | 79.7 ± 15 | 79.3 ± 15 | 82 ± 13 | 0.002** |
| Weight (kg) | 66.9 ± 12 | 66.4 ± 11 | 71.5 ± 18 | 63.5 ± 12 | 62.7 ± 12 | 68.3 ± 11 | 0.012*** |
| Belt circumference (cm) | 92.3 ± 14 | 91.7 ± 13 | 97.4 ± 20 | 90.0 ± 13 | 90.5 ± 13 | 86.7 ± 11 | 0.1 |
| BMI (kg/m[2)] | 26.8 ± 4 | 27 ± 4 | 25.1 ± 4 | 25.8 ± 5 | 26.0 ± 5 | 24.5 ± 4.4 | 0.05 |
| FBS (mg/dL)* | 99.5 ± 40 | 100.1 ± 43 | 95.1 ± 14 | 88.6 ± 28 | 89.1 ± 29 | 85.5 ± 19 | 0.001*** |
| TG (mg/dL)* | 105 ± 36 | 133.4 ± 98 | 113 ± 61 | 160.3 ± 99 | 162.0 ± 97 | 149 ± 13 | 0.005** |
| Cholesterol (mg/dL)* | 202 ± 180 | 195 ± 41 | 176 ± 35 | 128.2 ± 41 | 195.7 ± 41 | 185 ± 36 | 0.06 |
| LDL-C (mg/dL)* | 117 ± 36 | 116.2 ± 37 | 122 ± 23 | 126.3 ± 35 | 129.2 ± 26 | 122 ± 31 | 0.005** |
| HDL-C (mg/dL)* | 46.6 ± 11 | 47.7 ± 11 | 38.6 ± 11 | 34.9 ± 12 | 35.3 ± 13 | 32 ± 10 | 0.0001 |
| Smoking (%) | 6.9% | 4.9% | 21.4% | 18.4% | 16.2% | 31.9% | 0.001** |
| MetS (IDF)(%) | 30.8% | 41.5% | 28.6% | 34.2% | 57.8% | 29% | 0.005** |
| MetS (NCEP–ATP III)(%) | 45.2% | 37.7% | 28.6% | 53.8% | 51% | 21.7% | 0.0001** |

* Patients treated with hypotensive, hypoglycemic, or lipid-lowering agents were included.

** Significantly higher in the control group.

*** Significantly higher in the case group.

### Biochemical and Drug History of RA Patients

RA patients in our study were categorized into four and two groups according to the DAS28ESR classification, in order to find the impact of MetS on disease activity or vice versa. The mean duration of the disease was 5.5 ± 5.2 years. The patients had a mean ESR of 20 (13–37) mm/h, C-reactive protein (CRP) 20 (0–40) mg/L, rheumatoid factor (RF) 20 (0–40) units/mL, and VAS 47.2 ± 27.7 mm. Antibodies to cyclic citrullinated peptides (anti-CCPs) were positive in 70% and RF was positive in 79% of this population. Table 3 shows the classifications, numbers, and percentages of patients in each group, as well as correlations between MetS and disease activity. The patients' drug histories included prednisolone with an average dose of 5.5 ± 3.6 (n = 108), and 3–6 mg/kg of hydroxychloroquine and methotrexate with an average dose of 7.9 ± 5.7 per week (n = 90). In addition, 32 patients were being treated with sulfasalazine or azathioprine, three with rituximab, and two with infliximab at the time of study. All patients received supplementary calcium and vitamin D. Thirteen patients had early RA (<6 months past the beginning of the RA).

**TABLE 2**
**Frequency and Statistical Differences of Metabolic Components in Men and Women of Patient and Control Groups**

| MetS Components | Female (F) | | | Male (M) | | |
|---|---|---|---|---|---|---|
| | Patients (n) (%) | Controls (n) (%) | *p* Value† | Patients (n) (%) | Controls (n) (%) | *p* Value† |
| FBS ≥ 110 mg/dL* | 17 (18.9) | 35 (8.2) | 0.002** | 2 (14.3) | 2 (3.9) | 0.07 |
| TG ≥ 150 mg/dL*** | 24 (26.4) | 191 (45) | 0.001*** | 3 (21.4) | 2 (3.9) | 0.3 |
| HDL-C < 50 mg/dL*** (F)<br>HDL-C < 40 mg/dL*** (M) | 62 (66) | 384 (89.7) | 0.000** | 6 (44.2) | 50 (72.5) | 0.06 |
| LDL-C ≥ 130 mg/dL*** | 26 (30.6) | 182 (44.1) | 0.02** | 4 (28.6) | 24 (36.4) | 0.7 |
| Cholesterol ≥ 200*** | 32 (35.6) | 176 (40.9) | 0.3 | 4 (28.6) | 18 (26.5) | 0.7 |
| Waist (cm) > 88 (F)<br>Waist (cm) > 102 (M) | 53 (56.4) | 237 (56.2) | 0.9 | 4 (28.6) | 6 (8.7) | 0.01* |
| BP ≥ 120/85 mmHg*** | 30 (30.6) | 213 (49.4) | 0.001** | 4 (28.6) | 33 (47.8) | 0.18 |

† Chi-square or Fisher's exact test.

* Patients treated with hypotensive, hypoglycemic, or lipid-lowering agents were included.

** Significantly higher in patients.

*** Significantly higher in controls.

**TABLE 3**
**Correlation between RA Disease Activity and Presence of MetS**

| DAS Classification | MetS (IDF Criteria)* | | MetS (NCEP–ATP III Criteria)* | |
|---|---|---|---|---|
| | Yes | No | Yes | No |
| DAS < 2.6 | 3 (7.7) | 13 (16) | 4 (8.8) | 13 (15.2) |
| 2.6 ≤ DAS < 3.2 | 4 (10.5) | 7 (8) | 5 (11.8) | 6 (7.6) |
| 3.2 ≤ DAS ≤ 5.1 | 14 (34.2) | 30 (38.7) | 13 (35.3) | 30 (36) |
| DAS > 5.1 | 20 (47.4) | 29 (37.3) | 15 (44.1) | 31 (39.2) |
| Total | 41 (100) | 79 (100) | 37 (100) | 83 (100) |
| *p* value** | 0.4 | | 0.7 | |

* Values are expressed as number (%).

** Chi-square or Fisher's exact test.

### Comparison of Prevalence of MetS in Case and Control Groups

The overall frequency of MetS was 30.8% in patients and 45.2% in controls. MetS had significantly lower prevalence in patients ($p = 0.005$). There was no significant difference between the prevalence of MetS in RA patients with ATP III and IDF criteria definitions ($p = 0.1$); however, in the control group, the ATP III–defined MetS was present at a significantly higher prevalence than when IDF criteria were applied ($p > 0.001$).

#### Distribution of MetS, Its Components, and Cardiovascular Traditional Risk Factors, According to Sex, in Patients and Controls

As shown in Table 1, features of MetS with its two definitions, including systolic and diastolic blood pressure, serum levels of TG, LDL-C, and also percentage of smokers, were significantly higher in healthy controls ($p < 0.05$); while weight, BMI, and FBS were higher in patients ($p < 0.05$). RA patients were more likely to have high HDL-C compared with controls. There was no significant difference in ESR, CRP, anti-CCP, and RF between patients with and without MetS. Table 2 reveals that only FBS ≥ 110 mg/dL or diabetes was present at a higher frequency in female patients, and waist circumference > 102 cm was higher in male patients in comparison to the age- and sex-matched controls. In this study, 10.8% of patients had a history of ischemic heart disease, 0.9% myocardial infarction (MI), 13.7% hypertension, 8.6% diabetes, and 6.5% a positive family history of RA in their first relatives. There was no significant difference regarding the percent of smokers, history of MI, hyperlipidemia, and early RA between patients with and without MetS; while diabetes ($p < 0.001$), history of established ischemic heart disease ($p = 0.03$), and hypertension ($p < 0.001$) were higher in patients with IDF-defined MetS. Only diabetes ($p < 0.001$) and hypertension ($p < 0.001$) frequency were higher in patients with NCEP–ATP III MetS.

#### Correlation of the DAS28ESR with MetS and Metabolic Components

Table 3 shows that there was no significant difference in the frequency of two MetS definitions among subgroups with different stages of disease activity. When we categorized patients into two groups of disease activity according to the DAS28, there was no significant difference in the frequency of MetS according to IDF ($p = 0.4$), NCEP–ATP III ($p = 0.8$), or both ($p = 0.8$) criteria between patients with DAS < 3.2 and DAS ≥ 3.2 (Chi-square test). On the other hand, there was no significant difference in the DAS28 between subgroups of patients stratified according to the existence of MetS — IDF definition: DAS = 4.5 ± 1.7 in patients without MetS, DAS = 4.8 ± 1.6 in patients with MetS ($p = 0.3$); NCEP–ATP III definition: DAS = 4.7 ± 1.6 in patients without MetS, DAS = 4.7 ± 1.6 in patients with MetS ($p = 0.7$); and both definitions: DAS = 4.5 ± 1.7 in patients without MetS, DAS = 4.7 ± 1.6 in patients with MetS ($p = 0.7$).

Table 4 illustrates that the number of individual components of MetS was significantly increased with increasing age, BMI, waist circumference, and weight, while it decreased with increasing dose of prednisolone and hydroxychloroquine. Among other important demographics, the percentage of diabetic ($p = 0.001$) and hypertensive ($p = 0.001$) (Mann-Whitney test) patients was significantly increased in parallel with the number of metabolic components. On the other hand, the male to female ratio, history of MI, positive family history of CVD, and the percentage of smokers did not change with increasing number of metabolic components.

This study revealed that waist circumference, systolic and diastolic blood pressure, FBS, TG, LDL-C, and HDL-C had no correlation with DAS. Only age (r = 0.2, $p = 0.006$), positively, and weight (r = -0.19, $p = 0.04$), negatively, were found to be significantly correlated with DAS in the Spearman's correlation test.

#### Multiple Logistic Regression Analysis

Among important demographics of the patients, including age, BMI, weight, DAS, VAS, and dosage of drugs used to treat patients (including prednisolone, hydroxychloroquine, and methotrexate), only BMI, weight, and age showed a significant difference between patients with and without MetS (*t*-test). Multiple logistic regression analyses were conducted with BMI, age, and weight as confounding factors. The analyses identified age (β = 0.067, OR = 1.069; 95%CI, 1.026, 1.114; $p = 0.002$) and BMI (β = 0.196, OR = 1.21; 95% CI, 1.07, 1.37; $p = 0.001$) as significant predictors of MetS.

**TABLE 4**
**Correlation between Existence of Metabolic Components and Important Demographics of RA Patients**

| Demographics | MetS Component (n = 0) | MetS Component 1–2 | MetS Component ≥ 3 | *p* Value |
|---|---|---|---|---|
| Age | 41.7 ± 12 | 42.3 ± 14 | 52.9 ± 8.8 | 0.000† |
| DAS | 4.5 ± 1.6 | 4.6 ± 1.7 | 4.7 ± 1.6 | 0.9† |
| VAS | 45 ± 24 | 44.5 ± 28 | 53 ± 27 | 0.3† |
| Disease duration (years) | 6.4 ± 6.9 | 5.5 ± 4.1 | 5.1 ± 4.7 | 0.9† |
| Prednisolone (mg/day) | 6.7 ± 2.8 | 4.6 ± 3.1 | 6.1 ± 3.9 | 0.02†† |
| Methotrexate (mg/week) | 8.9 ± 4.5 | 8.8 ± 4.5 | 8.5 ± 6.9 | 0.9†† |
| Hydroxychloroquine (mg/day) | 286.9 ± 157 | 158 ± 174 | 212 ± 186 | 0.01†† |
| BMI (kg/$m^2$) | 26.1 ± 3.2 | 25.8 ± 4.9 | 28.8 ± 4.4 | 0.006 |
| Belt (cm) | 79.2 ± 6.3 | 91.9 ± 14 | 100.7 ± 4.4 | 0.000 |
| Weight (kg) | 64.7 ± 10 | 64.7 ± 12 | 71.7 ± 13 | 0.02 |
| ESR (mm/H) | 21.5 ± 16 | 32 ± 27 | 27 ± 17 | 0.2 |

Values are expressed as mean ± SD.

† One way ANOVA test

†† Kruskal-Wallis test

The association between individual MetS components, BMI, weight, drug dosages (prednisolone, hydroxychloroquine, methotrexate), and the DAS28ESR was next assessed by linear regression analysis, adjusting for sex and age. We found an inverse correlation between BMI ($\beta$ = –0.08, $p$ = 0.02) and the DAS28ESR, whereas no relationship was observed with other components.

Multiple logistic regression analysis showed that the odds of disease activity > 3.2 in patients with BMI between 25 and 30 kg/$m^2$ (OR = 0.1, $p$ = 0.01) and BMI > 30 kg/$m^2$ (OR = 0.3, $p$ = 0.1) in comparison to BMI < 25 is 1/5 and 1/3, respectively. Therefore, most patients with active disease in this study had normal or low BMI.

To evaluate the simultaneous effects of BMI and RA on MetS, multiple logistic regression was conducted. It illustrated that BMI ($\beta$ = 0.2, $p$ < 0.001) and RA ($\beta$ = –0.8, $p$ < 0.001) independently influenced MetS. As shown above, RA has negative effect on MetS.

### DISCUSSION

In this study of middle-aged individuals, MetS was found to be more common in the normal population (45.2%) than in RA patients (30.8%). The current study suggests that two definitions of MetS, namely IDF and ATP III criteria, do not differ in their estimation of MetS prevalence in patients. We found no correlation between the RA disease activity index according to the DAS28ESR and MetS existence with both its definitions. The current study showed that only the average of FBS or diabetic patients, weight, and BMI is higher in RA patients in comparison to the sex- and age-matched normal population. Two other important results of this study are that patients with active RA have lower BMI and lower prevalence of MetS.

According to previous studies on the prevalence of MetS in RA patients, its rate ranges from 14 to 45%[9,27,28]. Toms et al.[22] confirmed that the prevalence of MetS in RA depends on the definition used. In their study, IDF criteria showed the highest prevalence of MetS (45.3%), the NCEP–ATP III 2001 showed a prevalence of 38.3%, which was near to the IDF criteria, and the European Group for

Insulin Resistance (EGIR) estimated the lowest prevalence for this condition (12%) in RA patients. We have also shown here that in RA patients, the prevalence of MetS with IDF and ATP III criteria was 30.8 and 45.2%, respectively. These two criteria did not differ significantly in estimating the prevalence of MetS in RA patients. On the other hand, in the sex- and age-matched general population, ATP III estimated a significantly higher prevalence of MetS. In addition to the present study, several other studies accentuated the independent effect of aging on the prevalence of MetS[9,22,29]. The difference between the prevalence of MetS in cases and controls in our study may stem from several reasons. The first is the lower age of our patients in comparison to those in the Karvounaris et al.[9] and Chung et al.[28] studies. Since older patients may have more disabilities leading to sedentary life style, compared to the age-matched normal population, we assume that the lower prevalence of MetS in our patients compared to controls may result from recruitment of younger patients with less disabilities. Second, in this study, a substantial proportion of patients were treated with hydroxychloroquine. It is clear that this drug acts as a lipid profile modulator and affects blood glucose levels, and therefore impacts on the definition of MetS[20,21]. It has been shown in this research that the least number of metabolic components correlates with the higher dose of this medication. Aside from the hydroxychloroquine, most of these patients were treated with methotrexate, which may also reduce the likelihood of MetS in RA[22]. Third, recent research reports indicate that glucocorticoids, at the low dose routinely advised for the treatment of RA, can increase HDL-C without increasing LDL-C or TG[17]. Several mechanisms have been used to explain the improvement of lipid profiles associated with low-dose glucocorticoids, such as activation of lipoprotein lipase and the decreased activity of hepatic TG lipase[17,30,31]. In addition, reduced activity of cholesterol ester transfer protein (CETP) has been reported in patients with early RA after immuno-intervention with methotrexate and prednisone[32]. Regarding the function of CETP[17,32,33], its reduced activity may be responsible for a rise in HDL-C. With respect to the aforementioned impacts of antirheumatoid drugs on the MetS components, discrepancies between current findings and those of previous studies may be due to the differences in the type and/or dose of the treatment regimen used by the RA patients. Fourth, recruitment of the control group in this study was from public health care centers, where people attended for contraceptive advice or vaccination. Most of these people would not have taken particular care about their cardiovascular risk modifications, while behavior modification, as well as periodic control for hyperlipidemia and diabetes, may alter individual risk factors that comprise the MetS in RA. Apart from the reasons mentioned, one important study in our geographic region mentioned that in the Iranian population, the presence of MetS defined by either the NCEP–ATP III or IDF criteria fails to identify individuals with established angiographically defined CAD. Therefore, they offered that population-specific definitions are required[7]. Finally, differences in nutritional habits and genetic susceptibility among different populations may also affect the prevalence of MetS risk factors and account for at least part of the observed discrepancies.

Unlike most previous studies[9,28,34], we did not observe any association between RA disease activity and MetS in our study. Interestingly, we observed that BMI negatively correlates with DAS, which can be compared with the outcomes of the research conducted by Kaufmann et al.[35] and Escalante et al.[37]. They proposed that BMI can be a predictor of mortality or joint damage in RA. In addition, this issue could be a reflection of RA cachexia during active disease. Some researchers have proposed that patients with RA have higher BMI with more central obesity than BMI-matched normal people, and this kind of adipose distribution may reflect RA cachexia. Hence, central obesity may be a more important predictor of CVD than higher BMI[36]. However, in our study, the waist circumference had no difference between patients and controls. On the other hand, BMI was a better predictor for number of metabolic components in RA patients than waist circumference.

The current study was not without limitations. The major limitation is that the DAS28ESR, which was used in the present study, is only a single-point measure of disease activity, whereas for RA, this will vary over time. A single measure does not give a very effective estimation of disease activity overall, and this may be a reason for the lack of association between disease activity and presence of MetS. Therefore, using multiple markers could give a better estimation of disease activity and its association with MetS or BMI. As another limitation, most of our patients were being treated during the study. Therefore, the real impact of MetS on disease activity, as can be observed in naive patients with RA after a period of follow-up, was not assessed. Moreover, insulin resistance and accelerated atherosclerosis, the main outcomes of MetS, were not measured in this study. Hence, it is not clear that despite the lower frequency of MetS, the mentioned factors are also lower in these patients. Finally, control subjects of the present investigation were apparently healthy subjects recruited from public health care centers. Self-reporting might be inaccurate for diseases with diagnostic criteria that are not well established, such as MetS. In addition, the prevalence of MetS is very high in the Iranian population. With self-reporting, there is always the possibility of under-reporting, although this report is also present with medical report–assessed health.

Taken together, the findings of the present study suggest that the frequency of MetS may decrease significantly in RA, which is probably due to the aggressive treatment and life style modification advised to these patients. It was also found that MetS and RA disease activity do not correlate together. A hypothesis for the present findings may be that the IDF and/or NCEP–ATP III criteria are not reliable criteria to diagnose MetS in Iranian patients with RA. Therefore, further research might explore the impact of other MetS definitions, such as those of the WHO and EGIR. In addition, as previously suggested[7], development of new and population-specific MetS definitions might be required for the Iranian population. Finally, while our results are in agreement with some recent studies, the beneficial effect of higher BMI in RA remains to be verified[35,37,38].

### ACKNOWLEDGMENTS

This study was financially supported by the Research Council of the Mashhad University of Medical Sciences, Mashhad, Iran.
